# Approach to suspected donor-derived infections

**DOI:** 10.3389/fped.2023.1265023

**Published:** 2023-10-04

**Authors:** Hannah Kinard Bahakel, Rebecca Pellet Madan, Lara Danziger-Isakov

**Affiliations:** ^1^ Division of Infectious Diseases, Cincinnati Children’s Hospital Medical Center, Cincinnati, OH, United States; ^2^ Division of Pediatric Infectious Diseases, New York University Langone Health, New York, NY, United States; ^3^ Department of Pediatrics, New York University Grossman School of Medicine, New York, NY, United States; ^4^ Department of Pediatrics, University of Cincinnati College of Medicine, Cincinnati, OH, United States

**Keywords:** pediatric, transplant, donor-derived infection, solid organ, donor screening

## Abstract

Prevention of donor-derived disease among pediatric solid organ transplant recipients requires judicious risk-benefit assessment. Comprehensive guidelines outline specific donor risk factors and post-transplant monitoring strategies to prevent and mitigate transmission of HIV, hepatitis B, and hepatitis C. However, elimination of unanticipated donor-derived infections remains challenging. The objectives of this review are to (1) define risk of anticipated vs. unanticipated disease transmission events in pediatric solid organ transplant recipients; (2) discuss donor presentations that confer greater risk of unanticipated disease transmission; (3) develop a matrix for consideration of donor acceptance; and (4) discuss limitations and future directions for donor screening. Although solid organ transplant confers inherent risk of infection transmission, the risk of significant disease transmission events may be mitigated by a comprehensive approach including donor assessment, consideration of recipient need, post-transplant monitoring, and early intervention.

## Introduction

In 2010, the Centers for Disease Control and Prevention (CDC) reported two kidney transplant recipients who acquired *Balamuthia mandrillaris* granulomatous amebic encephalitis from a single, four-year-old organ donor who had signs and symptoms of meningoencephalitis and who was incorrectly presumed to have died following influenza A-associated acute disseminated encephalomyelitis (1). One kidney recipient died secondary to neurologic complications, one kidney recipient survived but with significant neurologic impairment, and two pediatric organ recipients from the same donor survived following early, empiric therapy targeted against *B. mandrillaris.* This case underscores the challenges of recognizing transmissible diseases among organ donor candidates. It also highlights the challenges of diagnosing donor-derived infections in recipients and facilitating critical communication between transplant centers and organ procurement organizations to identify and treat impacted recipients. The following review will discuss these challenges and provide a framework for pediatric transplant specialists.

Solid organ transplantation (SOT) remains the definitive therapy for children with organ failure. Donor-derived infections, while rare, are an inherent risk of solid organ transplantation and may result in significant morbidity and mortality (2, 3). It is therefore essential to minimize unanticipated disease transmission events, while simultaneously maximizing utilization of the limited number of donor organs. Pre-transplantation screening of potential organ donors and recipients can mitigate the risk of common donor-derived infections (2). A number of comprehensive guidelines outline strategies for the detection and prevention of common pathogens that may be transmitted through transplantation, and organ procurement organizations and transplant centers typically employ rigorous screening protocols for potential donors and recipients. While these conventional screening strategies are effective in most cases, unanticipated transmission events can still occur and may be challenging to recognize (4).

## Expected transmission events

Donor-derived infections can be categorized into two groups: “expected” and “unexpected.” Expected transmission events occur when a known infection is identified in a potential donor, with the recognized potential for transmission to one or more recipients. The most common infections in this category are viral pathogens such as cytomegalovirus (CMV), Epstein-Barr virus (EBV), and hepatitis B and C. Bacterial, fungal, and parasitic transmissions can also occur as the result of infection or colonization of the donor organ, contamination during organ recovery, or transmission through the bloodstream during the transplantation process (5). Preventative strategies such as targeted antimicrobial prophylaxis, perioperative infection prevention measures, and post-transplant surveillance can minimize the risk and impact of expected disease transmission. The Centers for Disease Control and Prevention (CDC), the Organ Procurement and Transplantation (OPTN), and the American Society of Transplantation (AST) provide detailed recommendations for screening for human immunodeficiency virus (HIV), hepatitis B and C, CMV, and other relevant donor-derived pathogens (2).

## Unexpected transmission events

Unexpected disease transmission events occur when pathogens are not detected in an organ donor prior to organ recovery or are transmitted to SOT recipients despite routine screening and preventative measures. Unexpected disease transmission events are reported to occur in fewer than 0.2% of SOT recipients but can result in significant consequences for the recipient (4, 6, 7). When an unexpected transmission event is suspected, prompt diagnosis and treatment, as well as effective communication with public health authorities and the OPTN, are essential.

## When to suspect donor-derived infection

Early recognition of donor-derived infection is critical for appropriate management. If a SOT recipient develops signs or symptoms of an unusual infection shortly after transplantation, a donor-derived infection should be considered. Recipients with donor-derived infections typically manifest within the first month post-transplant; however, it is important to note that time to presentation may vary significantly depending on the type of infection that is transmitted, the net state of immunosuppression of the recipient, and the type of organ transplanted (8).

Donor-derived neurotropic viruses may result in central nervous system (CNS) disease in as little as one week following transplant, whereas tuberculosis, parasitic infections, and fungal infections may take several months to manifest. Interestingly, donor-derived hepatitis B in liver transplant recipients is often reported more than a year post-transplant (9). A case in which invasive aspergillosis was transmitted from a single, asymptomatic donor to three organ recipients underscores the variability of time to donor-derived disease presentation. While the two kidney transplant recipients in this case became symptomatic within three weeks of transplantation, the heart transplant recipient did not present until approximately six months post-transplant (10). Transplant clinicians should therefore consider the possibility of a donor-derived infection in SOT recipients presenting with an unexplained febrile illness, leukocytosis, or other signs of occult infection, even if symptoms manifest after the first month post-transplant (8). If multiple organ recipients from a single donor develop similar infectious complications, a donor-derived infection should be suspected, as this suggests transmission from the donor as the common source of infection. As discussed in greater detail below, it is critical for transplant centers to promptly report even suspected donor-derived infections, as this may allow for earlier diagnosis and intervention for affected organ recipients at other centers. Clusters of infections have been reported in multiple recipients who have received organs from a single donor, including fungal infections, herpes simplex virus (HSV), lymphocytic choriomeningitis virus (LCMV), rabies virus, HIV, and hepatitis C (11).

## Approach to suspected donor-derived infection

Certain donor presentations may confer a greater risk of disease transmission. Screening donors at potentially increased risk for transmission of infection may require evaluation on an individual basis; published guidelines exist to identify donors with behaviors that increase the risk for unanticipated HIV, hepatitis B, and hepatitis C acquisition (12). These include donors with a history of intravenous drug use, unprotected sexual activity, multiple sexual partners, or recent incarceration within 30 days of potential donation. As donor history may be limited for several reasons and pre-transplant testing may occur during window periods between infection and test positivity, OPTN policy mandates post-transplant testing of all solid organ transplant recipients for HIV, hepatitis B, and hepatitis C (13). The most current policy requires testing at least 28 days but no later than 56 days post-transplant on all recipients. Due to delayed hepatitis B transmission, liver transplant recipients must additionally have repeat hepatitis B testing between 335 and 395 days post-transplant.

Beyond HIV and viral hepatitis, there are unfortunately fewer resources available to uniformly identify donors at greater risk to transmit an unanticipated infection (13). Clinicians must therefore maintain a high index of suspicion for donor-derived infection, and careful evaluation of donor characteristics, including prior travel and region of residence, should be undertaken when a recipient presents with a clinical syndrome concerning for donor-derived disease. Donor characteristics to consider in the evaluation of a suspected donor-derived infection include the presence of a pre-existing infection. Donors with a known or suspected active infection at the time of donation, such as pneumonia, urinary tract infection, bloodstream infection, or an endemic infection such as *Coccidioides* may transmit the infectious agent to the recipient (12, 14–16). An estimated 5% of organ donors have bacteremia at the time of donation (17). However, targeted antimicrobial prophylaxis for recipients is highly effective in preventing disease transmission to the recipient.. Recent data in adults indicate that donor bacteremia is not negatively associated with graft or other adverse recipient outcomes and is typically not a contraindication to organ recovery and transplantation (18–20).

More recently, emergence of multi-drug resistant (MDR) organisms has posed a significant threat to SOT recipients who are exposed to these organisms through transplantation. Transplantation from donors who are colonized or infected with MDR gram-negative organisms has resulted in fatal post-transplant infection in SOT recipients despite antimicrobial treatment (21, 22). Risk of transmission may be related to the site of infection and organ transplanted. For example, donors with infection or colonization of the transplanted organ are considered higher risk for transmission. Receipt of an organ from a non-bacteremic donor with colonization of a non-transplanted organ, however, is considered lower risk of transmission. One study of adult SOT recipients reported the use of organs from donors with colonization or infection with MDR organisms with no transmission events in high-risk recipients who received appropriate antibiotic therapy (20). In addition, the detection of MDR organisms in donor respiratory cultures is not predictive of donor-derived infection in non-lung solid organ recipients and typically does not require targeted prophylaxis (22). Although MDR gram-negative colonization or infection in a donor is not an absolute contraindication to organ recovery and transplantation, it does require a cautious, case-by-case analysis of risks and benefits, as well as discussion regarding potential utility of antimicrobial prophylaxis. Some experts recommend that acceptance of an organ from a donor colonized with an MDR organism be contingent upon the existence of at least one antimicrobial agent with *in vitro* activity that could be administered to the recipient.

Despite laboratory screening of potential donors, transmission of respiratory viral infections from donor to recipient does occur. The risk of donor-derived respiratory viral infection is highest among lung transplant recipients (23–25). While donor-derived influenza A, influenza B, and SARS-CoV-2 infection have been documented in lung transplant recipients and have been associated with increased morbidity and mortality, data indicate excellent short-term outcomes for non-lung SOT transplant recipients from SARS-CoV-2 and influenza-positive donors (26–28).

## Clinical syndromes that should raise suspicion for donor-derived infection

Although donor-derived infections may present with non-specific signs such as altered mental status or fever, several post-transplant clinical syndromes should raise suspicion for specific donor-derived infection (29). When observed shortly after transplantation, signs or symptoms of an infection should prompt a thorough evaluation to determine if the infection originated from the donor. Development of meningitis, encephalitis, or other neurologic syndrome in an organ recipient should raise suspicion for donor-derived disease. Guidance for recognizing central nervous system infections in potential organ donors is available from The Organ Procurement and Transplantation Network (OPTN) Ad Hoc Disease Transmission Advisory Committee (DTAC) (13). These guidelines urge a cautious approach in considering transplantation of organs from donors with meningoencephalitis of unknown etiology (13). Meningoencephalitis has a variety of bacterial, viral, fungal, and protozoal causes, and case reports have documented transmission of West Nile virus (WNV), rabies virus, LCMV, *Cryptococcus gattii*, and *B. mandrillaris* through organ transplant (30). While routine screening of all donors for pathogens responsible for meningoencephalitis is not performed, potential organ donors with signs and symptoms of meningoencephalitis should undergo a comprehensive evaluation for infectious causes, with special consideration for opportunistic infection when the donor is from an endemic area, has recent potential exposures, or is immunocompromised secondary to medication and/or chronic illness (31, 32). For example, OPTN/DTAC has documented transmission of cryptococcosis from a donor with unrecognized meningoencephalitis (32). Recipient centers should be aware that many infections that result in meningoencephalitis, such as rabies, arboviruses, and LCMV, require specialized testing that may not result prior to organ transplantation. Current practices for screening organ donors for WNV vary by organ procurement organization (OPO). Some OPOs elect to screen all donors, while some report performing screening seasonally when WNV transmission is more likely to occur. Much uncertainty remains regarding the utility and effectiveness of screening organ donors for WNV (33).

When considering transplantation of organs from donors with meningoencephalitis of unknown etiology, centers should also consider that empiric therapy or prophylaxis for recipients is not available for many of the most transmissible and fatal (albeit less common) causes of non-bacterial meningoencephalitis, such as LCMV and arboviruses. Thus, centers should exercise extreme caution in transplanting organs from donors with meningoencephalitis of unknown etiology, and recipients who develop meningoencephalitis require aggressive evaluation. Donor-derived meningoencephalitis typically manifests within the first few weeks to months after transplantation; therefore if a SOT recipient develops CNS symptoms during the early post-transplant period, the possibility of donor-derived meningoencephalitis should be considered (4). Thorough diagnostic evaluation to identify the causative agent should be pursued and in collaboration with local public health authorities, who can offer thorough and expedited testing.

Donor-derived pneumonia most often occurs after lung transplantation but has been transmitted through transplantation of other solid organs as well. Typical symptoms that should raise suspicion for pneumonia include fever, dyspnea, and radiographic evidence of lung infiltrates. However, because transplant recipients are immunosuppressed, they may have atypical clinical presentations (34). Epidemiology, environmental exposures, and geographic location of the donor may influence the etiology of donor-derived pneumonia and increase the risk for certain types of endemic fungal infections or *Mycobacterium tuberculosis* (TB) infection (35). Several cases of donor-derived pulmonary TB have been reported in solid organ transplant recipients. A meta-analysis of more than 2,000 cases of TB in SOT recipients documented 23 cases of donor-derived TB, with most cases being in lung transplant recipients, though cases of donor-derived pulmonary TB were also reported in kidney, liver, and heart transplant recipients. Fever was the most common presenting symptom, though some cases were asymptomatic (34). Fever in a post-transplant patient with an at-risk donor should therefore prompt a thorough evaluation for TB disease.

Fungal infections such as coccidioidomycosis and histoplasmosis have been transmitted from donors who reside in specific geographical regions where these infections are endemic. Histoplasmosis is commonly found in the soil in certain regions of North America, particularly in the Ohio and Mississippi River valleys, whereas coccidioidomycosis is endemic to arid regions of southwest United States. It is estimated that donor-derived histoplasmosis occurs in approximately 1:10,000 transplants, and presents with symptoms of pneumonia, fever, sweats, weight loss, and lymphadenopathy (32). Similarly, several cases of donor-derived coccidioidomycosis in transplant recipients have been reported in both endemic and non-endemic areas and can present as fever and respiratory symptoms (32). With increased geographic sharing of organs, suspicion for donor-derived infection for recipients who receive organs from donors from endemic areas must be considered.

Similarly, parasitic infections such as *Strongyloides stercoralis* and *Toxoplasma gondii* can cause donor-derived infection and have wide geographic distributions (36, 37). Clinical syndromes attributed to Strongyloidiasis include acute infection, chronic infection, hyperinfection, and disseminated disease. Risk for donor-derived Strongyloidiasis is highest in the initial months following transplant, but has been described in the range of 7–33 weeks post-transplant (36). Symptoms include fever, respiratory symptoms, and abdominal symptoms as the larvae migrate through their life cycle, and mortality rate approaches 50%–70%. Prevention of donor-derived Strongyloidiasis currently includes targeted screening based on epidemiological risk factors; however, implementation of universal screening in the United States is anticipated with the recent OPTN policy change. Universal screening of all organ donors and recipients is recommended for *Toxoplasma*, as the morbidity and mortality of *Toxoplasma gondii* infection is unacceptably high (37). Toxoplasmosis symptom onset has been reported between two weeks and twenty years following transplant and most frequently occurs in heart transplant recipients. Clinical manifestations can be vague but can progress to involve multiple organ systems and include pneumonitis, myocarditis, meningitis, brain abscesses, and disseminated disease. A high index of suspicion for donor-derived endemic fungal and parasitic infections is therefore necessary to avoid diagnostic and therapeutic delays. Ideally, any known bacterial, fungal, or parasitic infection should be treated prior to transplantation (4). Living donors can undergo thorough infectious screening and treatment in advance of transplantation, but deceased donors may have limited pre-transplantation evaluation time with little ability to confirm resolution of infection.

## When to report a suspected donor-derived infection

Any suspected donor-derived infection should be reported promptly to the OPTN through the safety portal and to the responsible organ procurement organization, which will facilitate notification of other impacted recipient centers. Reporting a suspected donor-derived transmission event allows for timely investigation, appropriate management of potentially affected recipients, and implementation of preventative measures. Prompt reporting of a suspected donor-derived infection enables communication between transplant centers to allow for early detection of potential transmission events in other recipients from the same donor, which may mitigate or eliminate the risk of further transmission. In the United States, a passive reporting system is employed. Reported donor-derived transmission events are monitored by the OPTN Ad Hoc DTAC, with the goal of providing education and guidance to prevent future disease transmission events and to develop policies to enhance the safety of organ donation.

## Discussion

Solid organ transplantation confers an inherent risk of infection transmission, and mitigation of donor-derived infection remains a challenging yet critical aspect of transplant medicine. Pre-transplant screening of the potential organ donor and recipient, as well as post-transplant monitoring of the recipient, is essential to promptly identify any signs or symptoms of donor-derived disease. Despite these measures, the risk of infection transmission cannot be completely eliminated, and transplant clinicians should report any potential infections to minimize adverse outcomes and prevent future disease transmission.

## Data Availability

The original contributions presented in the study are included in the article/Supplementary Material, further inquiries can be directed to the corresponding author.
